# Supplementary material for: Iterative improvement in the automatic modular design of robot swarms
Source: PeerJ Comput Sci. 2020 Dec 7;6:e322. doi: 10.7717/peerj-cs.322 (PMC7924708; doi:10.7717/peerj-cs.322)
Supplement: Supplemental Information 3 [file peerj-cs-06-322-s003.zip › argos3/doc/api/standalone/a00573_source.html]

ARGoS: plugins/simulator/entities/led\_entity.h Source File


- Main Page
- Related Pages
- Namespaces
- Classes
- Files

- File List
- File Members

# plugins/simulator/entities/led\_entity.h

Go to the documentation of this file.

```
00001 
00007 #ifndef LED_ENTITY_H
00008 #define LED_ENTITY_H
00009 
00010 namespace argos {
00011    class CLEDEntity;
00012    class CLEDMedium;
00013 }
00014 
00015 #include <argos3/core/simulator/entity/positional_entity.h>
00016 #include <argos3/core/utility/datatypes/set.h>
00017 #include <argos3/core/utility/datatypes/color.h>
00018 #include <argos3/core/utility/math/vector3.h>
00019 #include <argos3/core/simulator/space/positional_indices/space_hash.h>
00020 #include <argos3/core/simulator/space/positional_indices/grid.h>
00021 
00022 namespace argos {
00023 
00024    class CLEDEntity : public CPositionalEntity {
00025 
00026    public:
00027 
00028       ENABLE_VTABLE();
00029 
00030       typedef std::vector<CLEDEntity*> TList;
00031       typedef CSet<CLEDEntity*> TSet;
00032 
00033    public:
00034 
00035       CLEDEntity(CComposableEntity* pc_parent);
00036 
00037       CLEDEntity(CComposableEntity* pc_parent,
00038                  const std::string& str_id,
00039                  const CVector3& c_position,
00040                  const CColor& c_color);
00041 
00042       virtual ~CLEDEntity() {}
00043 
00044       virtual void Init(TConfigurationNode& t_tree);
00045 
00046       virtual void Reset();
00047 
00048       virtual void Destroy();
00049 
00050       virtual void SetEnabled(bool b_enabled);
00051 
00058       inline const CColor& GetColor() const {
00059          return m_cColor;
00060       }
00061 
00069       inline const CColor& GetInitColor() const {
00070          return m_cInitColor;
00071       }
00072 
00078       void SetColor(const CColor& c_color);
00079 
00087       inline void SetInitColor(const CColor& c_color) {
00088          m_cInitColor = c_color;
00089       }
00090 
00091       virtual std::string GetTypeDescription() const {
00092          return "led";
00093       }
00094 
00104       void AddToMedium(CLEDMedium& c_medium);
00105 
00111       void RemoveFromMedium();
00112 
00118       inline bool HasMedium() const {
00119          return m_pcMedium != NULL;
00120       }
00121 
00127       CLEDMedium& GetMedium() const;
00128 
00129    protected:
00130 
00131       CColor m_cColor;
00132       CColor m_cInitColor;
00133       CLEDMedium* m_pcMedium;
00134 
00135    };
00136 
00137    /****************************************/
00138    /****************************************/
00139 
00140    class CLEDEntitySpaceHashUpdater : public CSpaceHashUpdater<CLEDEntity> {
00141 
00142    public:
00143 
00144       virtual void operator()(CAbstractSpaceHash<CLEDEntity>& c_space_hash,
00145                               CLEDEntity& c_element);
00146 
00147    private:
00148 
00149       SInt32 m_nI, m_nJ, m_nK;
00150 
00151    };
00152 
00153    /****************************************/
00154    /****************************************/
00155 
00156    class CLEDEntityGridUpdater : public CGrid<CLEDEntity>::COperation {
00157 
00158    public:
00159 
00160       CLEDEntityGridUpdater(CGrid<CLEDEntity>& c_grid);
00161       virtual bool operator()(CLEDEntity& c_entity);
00162 
00163    private:
00164 
00165       CGrid<CLEDEntity>& m_cGrid;
00166       SInt32 m_nI, m_nJ, m_nK;
00167 
00168    };
00169 
00170    /****************************************/
00171    /****************************************/
00172 
00173 }
00174 
00175 #endif
```

---

Generated on 10 Jul 2018 for ARGoS by 
 1.6.1
